# Examining the rural-urban divide in predisposing, enabling, and need factors of unsafe abortion in India using Andersen’s behavioral model

**DOI:** 10.1186/s12889-022-13912-4

**Published:** 2022-08-05

**Authors:** Margubur Rahaman, Puja Das, Pradip Chouhan, Kailash Chandra Das, Avijit Roy, Nanigopal Kapasia

**Affiliations:** 1 Department of Migration & Urban Studies, International Institute for Population Sciences (IIPS), Govandi Station Road, Deonar, Mumbai, 400088 India; 2 Department of Geography, University of Gour Banga, Malda, West Bengal 732103 India; 3 Department of Geography, Malda College, Malda, West Bengal 732101 India

**Keywords:** Unsafe abortion, Rural-urban, Andersen’s behavioral model, India

## Abstract

**Background:**

The prevalence of unsafe abortions significantly varies with geography; therefore, more research is needed to understand the rural-urban differences in unsafe abortion practices in India. The present study aims to explore the rural-urban differences in predisposing, enabling, and need factors of unsafe abortion in India.

**Methods:**

The present study used the fourth round of the National Family Health Survey (2015–16) and included the women aged 15–49 who terminated pregnancies by induced abortion during the 5 years prior to the survey (*N* = 9113) as the study sample. Descriptive statistics, bivariate chi-square significance test and multivariate logistic regression model were used to accomplish the study objectives.

**Results:**

The findings revealed that almost one-third of pregnancies were terminated through unsafe measures with sharp rural-urban contrast. The likelihood of unsafe abortions increases with decreasing women’s age and spousal level of education. Younger women in urban settings were more vulnerable to unsafe abortion practices. In rural settings, women with an uneducated spouse are more likely to have unsafe abortions (OR: 1.92). Poor households were more likely to undergo unsafe abortions, which were more common in rural settings (OR: 1.26). The unmet need for family planning was revealed to be a significant need factor for unsafe abortion, particularly in rural settings.

**Conclusion:**

Although abortion is legal, India’s high estimated frequency of unsafe abortions reveals a serious public health issue. Due to socio-economic vulnerability, unmet family planning needs, and a lack of awareness, significant numbers of women still practice unsafe abortions in India.

## Background

Unsafe abortion is a serious public health concern, adversely linked to reproductive health issues [1]. In the short term, unsafe abortion accelerates the risk of maternal mortality, haemorrhage, and post-abortion sepsis [2]. Consequently, the long-term risk includes the risk of ectopic pregnancy, premature delivery, miscarriages in subsequent pregnancies, sterility, and other psycho-physical disabilities [1, 3, 4]. In cognizance of prevailing repercussions, the World Health Assembly (2004) first endorsed unsafe abortion as a global reproductive health agenda and recommended the provision of safe abortion services [5]. The Sustainable Development Goals also included several targets to ensure sexual and reproductive health (SRH) rights and promote universal access to SRH services [6]. However, many countries in the lower-and-middle income countries have been facing unsafe abortion and associated health challenges due to the complexity of abortion laws, poor abortion healthcare services, and socio-economic poverty [7–10].

Worldwide, approximately 25 million abortions occur annually in unsafe settings, and Asian countries account for 50% of total unsafe abortions [11]. Moreover, India accounts for 6.5 million abortions, with two-thirds of abortions ending in unsafe settings [12, 13]. Furthermore, the National Family and Health Survey (2017) suggested that the prevalence of unsafe abortion is higher in rural India than in urban areas. In India, abortion is legal under the Medical Termination of Pregnancy (MTP) amendment bill (2020) for a wide range of medical and social reasons [14]. It should be performed under the guidance of trained providers [15]. However, a sizable portion of induced abortions continue to be carried out using unsafe methods [14] and are widespread among women with unwanted, close-spacing, and illegitimate pregnancies. Furthermore, a significant number of women perform sex-selective abortions through unskilled care providers, while sex-selective abortion is illegal in India by the MTP act (1971) [16, 17].

Socio-demographic and geographical divides in the practice of unsafe abortion are notable in India [18, 19]. Furthermore, many prior small-area-level studies suggest a significant difference in socio-demographic patterns of unsafe abortions between rural and urban areas [10, 20–22]. The proportion of teenage maternity, unwanted pregnancy, close-spacing pregnancy, and unmet need for family planning is more prevalent in rural areas, which upsurges the demand for induced abortions [22–24]. However, rural India’s high illiteracy, poverty, and poor healthcare infrastructure constrain access to safe abortion facilities [23]. The Government of India (GoI) implemented the National Rural Health Mission (NRHM) to improve grass-root level healthcare facilities. Despite the progress in healthcare coverage, there are still disparities based on place of residence [25]. In tune with circumstances, the study hypothesized that rural and urban India might have different socio-demographic patterns and determinants of unsafe abortion.

However, available literature has focused on the prevalence, pattern, and predictors of unsafe abortions [7, 18–20]. Nevertheless, the rural-urban gap in determinants of unsafe abortion practices, particularly in the Indian context using national representative data, remains to be explored. Therefore, sorting out the marginalized sub-groups between rural and urban settings is necessary to ease the inequalities more rationally. In line with this purpose, the present study aims to fill the gap through two central questions: Firstly, what are the socio-economic determinants of self-reported unsafe abortion in India? Secondly, how does the socio-economic determinant vary over geographical dynamics regarding rural and urban settings?

## Methods

### Data source and participants

The data was drawn from the fourth round of the National Family Health Survey (NFHS-4), conducted in 2015–16. The NFHS, an Indian version of the Demographic and Health Survey (DHS), provides consistent and reliable data on fertility, mortality, family planning, child nutritional status, reproductive and child healthcare utilization, and other related indicators [26]. The cross-sectional survey adopts a multistage stratified random sampling design in rural and urban areas. The NFHS-4 survey includes a total sample of 699,686 women; only the women aged 15–49 who terminated their pregnancies by induced abortion in the 5 years prior to the survey (*N* = 9113) were included in the present study.

### Outcome variable

The surveys collected information on abortion services using the following questions. In the pregnancy history section of the questionnaire, women were asked: *Have you ever had a pregnancy that miscarried, was aborted, or ended in a stillbirth?* The response was yes or no. Following that, a question was asked to the women who responded yes: *When did the last such pregnancy end?* The answers were: (a) the last pregnancy ended before January 2011, and (b) the last pregnancy ended in January 2011 or later. Lastly, a subsequent question was asked to the women who experienced only abortions, excluding miscarriage and stillbirth, of their last pregnancy in January 2011 or later: *who performed the abortions?* The responses were doctor, nurse, auxiliary nurse midwife (ANM)/lady health visitor (LHV), Dai, family member/relative/friend, self, and others. The responses were categorized into two categories in the present study: safe abortion (coded as 0)—which included induced abortions performed by doctors and nurses/ANM/LHV; and unsafe abortion (coded as ‘1’)—which included induced abortions performed by anybody other than skilled care providers [27].

### Explanatory variables

**Fig. 1:**
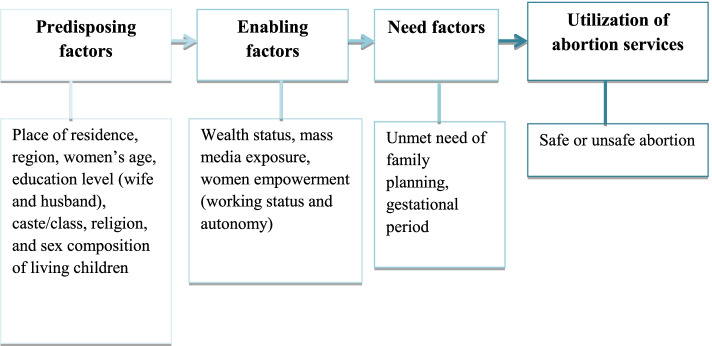
Conceptual framework of factors of unsafe abortion using Anderson’s behavioural model [29, 30]

Andersen’s behavioral model of healthcare utilization is a multilevel model that includes individual and contextual determinants of healthcare utilization [28, 29] and has been used as a conceptual framework in the present study. Using Andersen’s behavioral model of healthcare service utilization, the predictors were divided into three groups in the current study: predisposing, enabling, and need factors (Fig. 1). Predisposing factors included the place of residence (urban, rural), geographical region (north, central, east, northeast, south, and west), women’s age (15–19, 20–24, 25–29, 30–34, 35–39, 40+ years), women’s education level (no education, primary, secondary, or higher), husband’s education level (no education, primary, secondary, or higher), caste/class (general, other backward classes [OBCs], scheduled tribes and scheduled castes [STs/SCs]), religion (Muslim, non-Muslim), and sex composition of living children (no child, daughter only, son only, both) [7, 19]. Enabling factors included maternal wealth status (rich, middle, poor), mass media exposure (yes, no), working status (yes, no), and autonomy (yes, no) [14, 24, 29]. Need factors included the unmet need for family planning (yes, no) and gestational period (8 weeks, 9–12 weeks, ≥13 weeks) [22, 29].

### Statistical analysis

Descriptive statistics were presented to understand the distribution of research participants. Further, the bivariate analysis with Pearson’s chi-square significant test was performed to demonstrate the patterns of unsafe abortions by background characteristics. Multivariate logistic regression was applied using “safe abortion practices” as the reference category to access the determinants of unsafe abortions. In multivariate models, three consecutive models were employed based on Andersen’s healthcare utilization model. First, we considered only predisposing factors (place of residence, geographical region, women’s age, and level of education, husband’s education level, caste/class, religion, sex composition of living children) in model 1; followed by enabling factors (wealth status, mass media exposure, working status, and autonomy) added in model 2; and finally, need factors (unmet need of family planning, and gestational period) have been included in model 3 to access the adjusted effects of selected explanatory variables. The *p* ≤ 0.05 threshold for determining variables in the multivariate logistic regression model was considered. Before doing the multivariate analysis, we used the variance inflation factor (VIF) to check for multicollinearity across explanatory variables and found no sign of an issue. The odds ratio (OR), with a 95% confidence interval, is used to present the regression results. STATA version 14.0 was used for all statistical analyses (StataCorp LP, College Station, TX, USA).

## Results

### Background characteristics of the sample

Table 1 presents the background characteristics of the study sample. The results show that more than half of the sample belonged to the south, central, and eastern regions, irrespective of place of residence. Most of the samples were from the middle reproductive age group (25–29 years) in rural and urban settings. The urban sample population was wealthier and more educated than their rural counterparts. In particular, the percentage of poor and illiterate women was almost two and five times higher in rural compared to urban counterparts. The percentage of uneducated spouses was also twofold higher in rural areas compared to urban counterparts. The unmet need for family planning was slightly higher in rural areas than urban ones.

**Table 1:** Descriptive characteristics of the study population by place of residence, NFHS-4 India, 2015–16

	India		Rural India		Urban India	
	n	%	n	%	n	%
**Region**						
South	1920	21.1	969	18.1	951	25.4
Central	2552	28.0	1716	32.0	837	22.3
Eastern	2001	22.0	1351	25.2	650	17.4
North-Eastern	531	5.8	422	7.9	109	2.9
West	1109	12.2	435	8.1	674	18.0
Northern	1001	11.0	475	8.8	526	14.0
**Women’s age**						
40+	420	4.6	286	5.3	134	3.6
35–39	1117	12.3	606	11.3	511	13.6
30–34	2116	23.2	1168	21.8	948	25.3
25–29	3115	34.2	1824	34.0	1291	34.5
20–24	2107	23.1	1300	24.2	807	21.6
15–19	237	2.6	182	3.4	54	1.5
**Caste/Class**						
General	2769	30.4	1377	25.7	1392	37.2
OBC	3882	42.6	2312	43.1	1570	41.9
STs/SCs	2462	27.0	1678	31.3	784	20.9
**Women’s education**						
Secondary/Higher	6150	67.5	3243	60.4	2908	77.6
Primary	1177	12.9	791	14.7	386	10.3
No education	1786	19.6	1333	24.8	453	12.1
**Religion**						
Non-Muslim	7665	84.1	4705	87.7	2960	79.0
Muslim	1448	15.9	662	12.3	786	21.0
**Husband’s Education**						
Secondary/Higher	1240	13.6	688	12.8	552	14.7
Primary	213	2.3	141	2.6	72	1.9
No education	191	2.1	148	2.8	43	1.2
Missing	7470	–	4390	–	3080	–
**Sex composition of children**						
No child	899	9.9	484	9.0	415	11.1
Daughter Only	2587	28.4	1504	28.0	1083	28.9
Son Only	1851	20.3	955	17.8	895	23.9
Both	3776	41.4	2423	45.2	1353	36.1
**Wealth Status**						
Rich	4307	47.3	1499	27.9	2808	75.0
Middle	1971	21.6	1350	25.2	620	16.6
Poor	2836	31.1	2518	46.9	318	8.5
**Exposure to mass media**						
Yes	6289	69.0	3262	60.8	3027	80.8
No	2824	31.0	2105	39.2	720	19.2
**Women’s working status**						
Yes	353	21.4	223	22.6	130	19.5
No	1299	78.6	762	77.4	537	80.5
Missing	7461	–	4382	–	3079	–
**Women’s autonomy**						
Yes	7073	77.6	4084	76.1	2989	79.8
No	2040	22.4	1283	23.9	757	20.2
**Unmet need for family planning**						
No	7073	77.6	4396	74.6	2294	76.8
Yes	2040	22.4	1495	25.9	693	23.2
**Gestation week**						
≤ 8 weeks	1784	19.6	1011	18.8	773	20.6
9–12 weeks	1047	11.5	676	12.6	372	9.9
≥ 13 weeks	6282	68.9	3680	68.6	2601	69.4
**N**	**9113**	**–**	**5367**	**–**	**3746**	**–**

### The rural-urban difference in the prevalence of unsafe abortion

In India, the prevalence of unsafe abortion was found to be 7.2% higher in rural areas (30.3%) compared to its urban counterpart (23.1%) (Table 2). Geographical patterns showed that the rural-urban gap in the prevalence of unsafe abortion was highest in the central region (8.2%) and lowest in the west region (− 0.2%). Except for the early reproductive age group (15–19 years), the prevalence of unsafe abortion was higher in rural areas compared to urban areas among all peers. However, the prevalence of unsafe abortion was 20% higher among early reproductive women in urban settings (51.8%) than in their rural counterparts (31.8%). Regarding social groups, the prevalence of unsafe abortion was almost 10% higher among socio-economically backward women (SCs/STs/OBCs) who reside in rural areas than their urban counterparts. The rural-urban gap in the prevalence of unsafe abortion was also significant among women who had an only female child and more prevalent in rural settings. Women with an unmet need for family planning in rural areas reported more unsafe abortions than their urban counterparts.

**Table 2:** Rural-urban difference in unsafe abortion by selected background characteristics, NFHS-4 India, 2015–16

Explanatory variables	Unsafe abortions (%)			
	India	Rural India	Urban India	Rural-urban difference
**Total**	**27.3**	**30.3**	**23.1**	**7.2**
**Region**	***p*** **= 0.000**	***p*** **= 0.000**	***p*** **= 0.000**	
South	9.5	9.7	9.2	0.5
Central	42.2	44.9	36.7	**8.2**
Eastern	37.8	37.6	38.3	−0.7
North-Eastern	25.3	25.4	24.7	0.7
West	9.5	9.4	9.6	−0.2
Northern	23.5	22.1	24.6	−2.5
**Women’s age**	***p*** **= 0.006**	***p*** **= 0.011**	***p*** **= 0.017**	
40+	29.01	30.2	26.5	3.7
35–39	27.8	34.7	19.7	**15.0**
30–34	25.8	29.4	21.3	**8.1**
25–29	27.5	30.5	23.1	**7.4**
20–24	27.1	28.6	24.7	3.9
15–19	36.4	31.8	51.8	**−20**
**Caste/Class**	***p*** **= 0.000**	***p*** **= 0.011**	*p* = 0.568	
General	26.4	27.1	25.7	1.4
OBC	27.4	31.6	21.3	**10.3**
ST/SC	28.3	31.1	22.1	**9.0**
**Women’s education**	***p*** **= 0.000**	***p*** **= 0.000**	***p*** **= 0.001**	
Secondary/Higher	24.2	26.7	21.4	5.3
Primary	32.9	34.4	29.7	4.7
No education	34.6	36.7	28.5	**8.2**
**Religion**	*p* = 0.483	*p* = 0.054	***p*** **= 0.026**	
Non-Muslim	26.8	30.3	21.2	**9.1**
Muslim	30.4	30.6	30.2	0.4
**Husband’s Education**	***p*** **= 0.000**	***p*** **= 0.000**	*p* = 0.064	
Secondary/Higher	28.7	28.1	29.5	−1.4
Primary	34.8	34.3	35.8	−1.5
No education	42.3	42.3	42.4	−0.1
**Sex composition of children**	***p*** **= 0.000**	***p*** **= 0.000**	***p*** **= 0.000**	
No child	19.2	19.6	18.8	0.8
Daughter Only	27.2	30.2	22.9	**7.3**
Son Only	19.6	21.0	18.1	2.9
Both	33.2	36.2	27.8	**8.4**
**Household wealth status**	***p*** **= 0.000**	***p*** **= 0.000**	***p*** **= 0.015**	
Rich	21.1	21.8	20.8	1.0
Middle	28.2	28.2	28.1	0.1
Poor	36.2	36.5	33.7	2.8
**Exposure to mass media**	***p*** **= 0.000**	***p*** **= 0.000**	*p* = 0.053	
Yes	24.7	28.0	21.1	6.9
No	33.3	33.9	31.6	2.3
Women’s working status	*p* = 0.185	*p* = 0.592	*p* = 0.112	
Yes	33.7	33.2	34.6	−1.4
No	30.5	30.7	30.2	0.5
**Women’s autonomy**	*p* = 0.104	*p* = 0.062	*p* = 0.422	
Yes	28.6	27.7	29.7	−2.0
No	33.6	34.7	31.9	2.8
**Unmet need for family planning**	***p*** **= 0.000**	***p*** **= 0.000**	*p* = 0.668	
No	26.1	28.8	22.4	6.4
Yes	31.6	35.1	25.6	**9.5**
**Gestation week**	*p* = 0.045	*p* = 0.200	*p* = 0.072	
≤ 8 weeks	30.0	32.5	26.6	5.9
9–12 weeks	28.4	31.9	22.0	**9.9**
≥ 13 weeks	26.4	29.4	22.2	7.2

### Rural-urban differences in predisposing, enabling, and need factors of unsafe abortion

The likelihood of unsafe abortion was found to be 17% more likely in rural areas compared to urban counterparts in India (Table 3, model 1). Women’s age, geographical region, and sex composition of living children were significant predisposing factors to unsafe abortions in India, irrespective of place of residence. Household wealth status was found as a significant enabling factor for unsafe abortion in India, particularly in rural settings. The unmet need for family planning was found as a need factor for unsafe abortion in India, especially among rural dwellers (Tables 3, 4 and 5, model 3).

**Table 3:** Odds ratios of unsafe abortion from multivariate binary logistic regression models, NFHS-4 India, 2015–16

Explanatory variables	Model 1 OR (95% CI)	Model 2 OR (95% CI)	Model 3 OR (95% CI)
**Place of residence**			
Urban (Ref.)	1.00	1.00	1.00
Rural	1.17** (1.05,1.3)	1.08 (0.96,1.21)	1.08 (0.96,1.21)
**Geographical region**			
South (Ref.)	1.00	1.00	1.00
Central	5.82*** (4.67,7.25)	5.73*** (4.6,7.14)	5.75*** (4.61,7.16)
Eastern	5.57*** (4.43,7.01)	5.28*** (4.19,6.67)	5.30*** (4.2,6.69)
North-Eastern	2.28*** (1.8,2.89)	2.16*** (1.7,2.75)	2.16*** (1.7,2.74)
West	1.13 (0.81,1.58)	1.11 (0.79,1.55)	1.12 (0.8,1.57)
Northern	2.68*** (2.11,3.41)	2.74*** (2.15,3.48)	2.76*** (2.17,3.51)
**Women’s age**			
40+ (Ref.)	1.00	1.00	1.00
35–39	1.30* (1.01,1.66)	1.3* (1.01,1.67)	1.31*** (1.02,1.69)
30–34	1.39** (1.1,1.76)	1.4** (1.1,1.77)	1.42*** (1.12,1.8)
25–29	1.86*** (1.48,2.35)	1.88*** (1.49,2.37)	1.91*** (1.51,2.41)
20–24	2.16*** (1.68,2.76)	2.13*** (1.67,2.73)	2.15*** (1.68,2.76)
15–19	3.24*** (2.2,4.76)	3.07*** (2.09,4.53)	3.08*** (2.09,4.54)
**Caste/class**			
General (Ref.)	1.00	1.00	1.00
OBC	1.06 (0.94,1.2)	1.05 (0.93,1.19)	1.05 (0.93,1.19)
ST/SC	1.17*** (1.03,1.34)	1.14* (1.03,1.31)	1.15*** (1.02,1.31)
**Women’s education**			
Secondary/Higher (Ref.)	1.00	1.00	1.00
Primary	1.09 (0.95,1.27)	1.02 (0.88,1.19)	1.03 (0.88,1.19)
No education	1.11 (0.98,1.27)	1.01 (0.88,1.17)	1.02 (0.88,1.17)
**Religion**			
Non-Muslim (Ref.)	1.00	1.00	1.00
Muslim	0.95 (0.83,1.1)	0.95 (0.82,1.1)	0.96 (0.83,1.1)
**Husband’s Education**			
Secondary/Higher (Ref.)	1.00	1.00	1.00
Primary	0.99 (0.71,1.39)	0.98 (0.7,1.38)	0.99 (0.7,1.39)
No education	1.75*** (1.23,2.5)	1.69*** (1.18,2.42)	1.71*** (1.19,2.44)
**Sex composition of children**			
No living child (Ref.)	1.00	1.00	1.00
Daughter Only	1.63*** (1.32,2.02)	1.66*** (1.34,2.06)	1.62*** (1.30,2.01)
Son Only	1.15 (0.92,1.44)	1.18 (0.94,1.48)	1.15 (0.91,1.44)
Both	1.84*** (1.49,2.28)	1.85*** (1.49,2.3)	1.78*** (1.44,2.22)
**Wealth Status**			
Rich (Ref.)		1.00	1.00
Middle		1.12 (0.98,1.29)	1.12 (0.97,1.28)
Poor		1.23*** (1.06,1.42)	1.22*** (1.06,1.41)
**Exposure to mass media**			
Yes (Ref.)		1.00	1.00
No		1.09 (0.97,1.21)	1.08 (0.97,1.21)
**Women’s working status**			
Yes (Ref.)		1.00	1.00
No		0.93 (0.71,1.23)	0.93 (0.71,1.23)
**Women’s Autonomy**			
Yes (Ref.)		1.00	1.00
No		1.14 (0.9,1.44)	1.12 (0.89,1.42)
**Unmet need for family planning**			
No (Ref.)			1.00
Yes			1.19*** (1.06,1.33)
**Gestation week**			
≤ 8 weeks (Ref.)			1.00
9–12 weeks			0.93 (0.78,1.11)
≥ 13 weeks			0.91 (0.81,1.03)

*OR* odds ratio, *CI* Confidence interval, *Ref* Reference category

^***^p < 0.001

^**^p < 0.01

^*^p < 0.05

**Table 4:** Odds ratios of unsafe abortion from multivariate binary logistic regression models, NFHS-4 rural India, 2015–16

Explanatory variables	Model 1 OR (95% CI)	Model 2 OR (95% CI)	Model 3 OR (95% CI)
**Geographical region**			
South (Ref.)	1.00	1.00	1.00
Central	5.47*** (3.81,7.85)	5.54*** (3.86,7.95)	5.93*** (4.48,7.85)
Eastern	5.87*** (3.96,8.71)	5.78*** (3.88,8.6)	5.20*** (3.89,6.96)
North-Eastern	2.39*** (1.58,3.63)	2.23*** (1.46,3.41)	2.15*** (1.6,2.89)
West	1.5 (0.91,2.45)	1.45 (0.88,2.38)	0.89 (0.56,1.42)
Northern	3.22*** (2.17,4.77)	3.27*** (2.2,4.86)	2.58*** (1.9,3.51)
**Women’s age**			
40+ (Ref.)	1.00	1.00	1.00
35–39	1.18 (0.74,1.89)	1.18 (0.74,1.89)	1.35** (1.01,1.82)
30–34	1.28 (0.82,1.99)	1.27 (0.81,1.98)	1.48*** (1.12,1.95)
25–29	1.62*** (1.04,2.52)	1.64* (1.05,2.55)	2.03*** (1.54,2.67)
20–24	1.93*** (1.21,3.08)	1.90** (1.19,3.05)	2.27*** (1.69,3.04)
15–19	5.80*** (2.68,12.57)	5.45*** (2.49,11.92)	2.69*** (1.71,4.24)
**Caste/Class**			
General (Ref.)	1.00	1.00	1.00
OBCs	1.05 (0.85,1.3)	1.03 (0.84,1.27)	1.04 (0.89,1.22)
STs/SCs	1.08 (0.85,1.37)	1.06 (0.83,1.34)	1.16 (0.93,1.22)
**Women’s education**			
Secondary/Higher (Ref.)	1.00	1.00	1.00
Primary	1.1 (0.84,1.45)	1.04 (0.79,1.38)	1.01 (0.84,1.21)
No education	1.03 (0.79,1.33)	0.94 (0.72,1.24)	1.05 (0.88,1.24)
**Religion**			
Non-Muslim (Ref.)	1.00	1.00	1.00
Muslim	1.15 (0.92,1.43)	1.15 (0.92,1.44)	0.85 (0.7,1.03)
**Husband’s Education**			
Secondary/Higher (Ref.)	1.00	1.00	1.00
Primary	0.52 (0.26,1.02)	0.48 (0.24,0.96)	1.27 (0.85,1.89)
No education	1.42 (0.66,3.07)	1.41 (0.65,3.07)	1.92*** (1.27,2.90)
**Sex composition of children**			
No living child (Ref.)	1.00	1.00	1.00
Daughter Only	1.63*** (1.1,2.42)	1.64*** (1.10,2.43)	1.64*** (1.26,2.13)
Son Only	1.62* (1.08,2.43)	1.64* (1.09,2.47)	1.12 (0.74,1.3)
Both	2.22*** (1.5,3.3)	2.2*** (1.48,3.27)	1.66*** (1.28,2.16)
**Wealth Status**			
Rich (Ref.)		1.00	1.00
Middle		1.15 (0.9,1.45)	1.14 (0.96,1.35)
Poor		1.04 (0.77,1.4)	1.26*** (1.10,1.50)
**Exposure to mass media**			
Yes (Ref.)		1.00	1.00
No		1.22 (0.98,1.52)	1.03 (0.9,1.17)
**Women’s working status**			
Yes (Ref.)		1.00	1.00
No		0.68 (0.42,1.11)	1.03 (0.74,1.44)
**Women’s Autonomy**			
Yes (Ref.)		1.00	1.00
No		0.84 (0.55,1.28)	1.28 (0.97,1.7)
**Unmet need**			
No (Ref.)			1.00
Yes			1.20*** (1.05,1.37)
**Gestation week**			
≤ 8 weeks (Ref.)			1.00
9–12 weeks			1.00 (0.81,1.24)
≥ 13 weeks			0.95 (0.82,1.11)

*OR* odds ratio, *CI* Confidence interval, *Ref* Reference category

^***^p < 0.001

^**^p < 0.01

^*^p < 0.05

**Table 5:** Odds ratios of unsafe abortion from multivariate binary logistic regression models, NFHS-4 urban India, 2015–16

Explanatory variables	Model 1 OR (95% CI)	Model 2 OR (95% CI)	Model 3 OR (95% CI)
**Geographical region**			
South (Ref.)	1.00	1.00	1.00
Central	1.34* (1,1.8)	5.95*** (4.50,7.87)	5.64*** (3.92,8.11)
Eastern	1.44*** (1.09,1.9)	5.21*** (3.89,6.96)	5.90*** (3.96,8.80)
North-Eastern	1.98*** (1.51,2.61)	2.16*** (1.60,2.9)	2.21*** (1.45,3.38)
West	2.27* (1.7,3.04)	0.88 (0.56,1.41)	1.46 (0.89,2.41)
Northern	2.83*** (1.81,4.45)	2.57*** (1.89,3.49)	3.35*** (2.25,4.98)
**Women’s age**			
40+ (Ref.)	1.00	1.00	1.00
35–39	1.30* (1.01,1.66)	1.34* (1.01,1.81)	1.19 (0.75,1.91)
30–34	1.39** (1.1,1.76)	1.45*** (1.10,1.91)	1.27 (0.81,1.99)
25–29	1.86*** (1.48,2.35)	2.00*** (1.52,2.63)	1.65** (1.05,2.57)
20–24	2.16*** (1.68,2.76)	2.25*** (1.68,3.01)	1.89*** (1.18,3.04)
15–19	3.24*** (2.2,4.76)	2.70*** (1.72,4.25)	5.43*** (2.48,11.88)
**Caste/Class**			
General (Ref.)	1.00	1.00	1.00
OBC	1.06 (0.91,1.23)	1.04 (0.89,1.22)	1.04 (0.84,1.28)
ST/SC	1.21** (1.03,1.42)	1.17 (0.99,1.38)	1.05 (0.82,1.34)
**Women’s education**			
Secondary/Higher (Ref.)	1.00	1.00	1.00
Primary	1.08 (0.91,1.28)	1.01 (0.84,1.21)	1.05 (0.79,1.38)
No education	1.14 (0.98,1.33)	1.04 (0.88,1.23)	0.94 (0.71,1.24)
**Religion**			
Non-Muslim (Ref.)	1.00	1.00	1.00
Muslim	0.86 (0.71,1.04)	0.85 (0.7,1.03)	1.16 (0.92,1.45)
**Husband’s Education**			
Secondary/Higher (Ref.)	1.00	1.00	1.00
Primary	1.29 (0.87,1.93)	1.25 (0.84,1.86)	1.23 (0.24,1.95)
No education	1.98*** (1.32,2.97)	1.89*** (1.26,2.86)	1.12* (1.03, 1.19)
**Sex composition of children**			
No living child (Ref.)	1.00	1.00	1.00
Daughter Only	1.65*** (1.28,2.13)	1.68*** (1.3,2.18)	1.60** (1.07,2.38)
Son Only	0.99 (0.75,1.3)	1.01 (0.77,1.34)	1.19** (1.06,1.40)
Both	1.71*** (1.32,2.21)	1.73*** (1.33,2.25)	2.11*** (1.41,3.14)
**Wealth Status**			
Rich (Ref.)		1.00	1.00
Middle		1.14 (0.96,1.35)	1.15 (0.9,1.45)
Poor		1.27** (1.07,1.51)	1.05 (0.77,1.41)
**Exposure to mass media**			
Yes (Ref.)		1.00	1.00
No		1.04 (0.91,1.18)	1.22 (0.97,1.52)
**Women’s working status**			
Yes (Ref.)		1.00	1.00
No		1.04 (0.74,1.45)	0.68 (0.42,1.12)
**Women’s Autonomy**			
Yes (Ref.)		1.00	1.00
No		1.29 (0.98,1.72)	0.82 (0.53,1.25)
**Unmet need**			
No (Ref.)			1.00
Yes			1.18 (0.96,1.45)
**Gestation week**			
≤ 8 weeks (Ref.)			1.00
9–12 weeks			0.76 (0.55,1.06)
≥ 13 weeks			0.85 (0.68,1.05)

*OR* odds ratio, *CI* Confidence interval, *Ref*. Reference category

^***^p < 0.001

^**^p < 0.01

^*^p < 0.05

In particular, the likelihood of unsafe abortions was found to be more prevalent in the rural central and eastern regions than in the reference category, i.e., the southern part. The likelihood of unsafe abortions was found to be almost three times and five times more likely among the early reproductive age group (15–19 years) compared to the advanced reproductive age group (40+ years) in rural and urban areas, respectively (Tables 4 and 5, model 3). The likelihood of unsafe abortion was found to be significantly higher among women with only female children compared to only male or no children in India, irrespective of place of residence (Tables 3, 4 and 5, model 3). The women who belong to the poor wealth quintile in rural India were 26% more likely to access unsafe abortion services than their wealthy counterparts. Furthermore, the women with uneducated spouses were 92% more likely to access unsafe abortion services compared to higher educated spouses in rural settings (Table 4, model 3).

## Discussion

The prevalence and determinants of unsafe abortion were evaluated in the current study in both rural and urban settings in India. It has been demonstrated that women’s age, geographical region, sex composition of the living children, and husband’s level of education are important predisposing and need factors of unsafe abortion in India, both in rural and urban settings. Our findings support the commonsense predisposing factors to unsafe abortion, as concluded by Andersen in the Indian context [7]. However, household wealth status and unmet need for family planning were found to be enabling and need factors of unsafe abortion, particularly in rural India.

In line with prior studies in India [18, 31] and elsewhere [30], the present study found that unsafe abortion was more prevalent in rural than urban areas. A study by Banerjee & Andersen (2012) revealed that women residing in rural areas are often compelled to abort their pregnancies under untrained providers because of inadequate access to safe abortion procedures and a lack of knowledge about the location of safe abortion [7]. In support of these barriers, several prior studies [28, 32] have exhibited the lack of availability of primary health centres (PHCs) along with untrained health care providers in community health centres (CHCs) as the major hindrances to the utilization of safe abortion services in a rural setting. In addition, women in rural settings usually have a lower degree of autonomy and a high unmet need for family planning, which eventually leads them to access unsafe abortion practices [7, 24].

In line with Andersen’s behavioural model, our study also found geographical region as an important predisposing factor of healthcare utilization in the Indian context [7, 29]. Regarding geographical region, the prevalence of unsafe abortion was found to be significantly high in the rural central and eastern regions and may be aggravated due to the existence of socio-economic deprivation and the availability of limited healthcare services [19]. Both high socio-economic poverty and poor maternal healthcare services in rural central and eastern regions negatively affect safe health care service utilization, as suggested by many prior studies [33]. Andersen (1995) also mentioned that regional inequality in terms of the economy, healthcare facilities, and socio-cultural aspects shapes levels of healthcare utilization [29].

We found that early reproductive women had a higher likelihood of unsafe abortion than advanced reproductive women in both rural and urban India, which is consistent with research from Pakistan [34] and Nepal [30] but dissimilar with findings from Ghana [35]. Many previous studies suggested that a lack of knowledge about safe abortion service providers, the legal process of abortion, and the high unmet need for contraception for spacing increased the burden of unsafe abortion among early reproductive women in India and elsewhere [19–21, 34–36]. Furthermore, the likelihood of unsafe abortion was found to be significantly higher among early reproductive women (15–19 years), particularly in urban settings in India. Therefore, further study is needed to explore why the practice of unsafe abortion is so common among the early reproductive age group in metropolitan India.

There was no significant association between women’s level of education or autonomy and the risk of unsafe abortion practice in India. The result was similar to the previous study in India [19] but dissimilar to the findings from Nepal [30]. Furthermore, the insignificant association between women’s education and autonomy and choice of abortion care indicates that Indian women have limited power to make healthcare decisions. At the same time, the husband’s education was protective in performing induced abortions through unsafe methods in rural settings. The result suggested that all major health care decisions in a household are generally taken by the male member, and improving the level of education increases the utilization of safe health services in rural India [37].

Another interesting finding of our study is that the likelihood of unsafe abortions was found to be higher among women who had only daughters than among those who had only sons. This finding is consistent with several previous studies [18, 38]. The possible explanation for the prevailing variation lies in the practice of sex-selective abortions among women with only daughters, which often remained clandestine and were performed in unsafe settings [39, 40]. However, further study is needed to explore the patterns of unsafe abortion by region with high fertility and low sex ratio to understand better the association between sex-selective abortion or unwanted birth abortions and the risk of unsafe abortion practice.

Among the need factors, the unmet need for family planning was found as a significant determinant of unsafe abortion in India; the result is consistent with prior studies in Ghana [41]. Furthermore, women with an unmet need for family planning are more likely to perform unsafe abortions in rural settings. The unmet need for family planning is positively associated with unintended pregnancy, as suggested by many previous studies [18, 21, 41]. Most unintended pregnancies have been terminated through unsafe methods to minimize the cost of abortion and sidestep the legal procedure of induced abortion in India [24, 42].

In this study, household economic status was found as a function of healthcare decisions in India, particularly in rural settings; the findings support Andersen’s behavioral model. The socio-economically deprived individuals in rural settings mainly sought treatment from untrained healthcare providers [33]. As a result, women from socio-economically disadvantaged groups in rural settings who wanted to have an induced abortion typically sought untrained healthcare professionals.

### Policy implications

Our current study’s findings reveal some policy implications. First, an in-depth investigation into the reasons for unsafe abortions in high-focus regions in India is required. Second, safe abortion facilities at the village or ward level are required to ensure service availability and accessibility on the ground level. Third, it could be possible to prevent unsafe abortion by enhancing reproductive health services and reducing unintended pregnancies. Finally, a collaborative negotiation among community-level religious, political, administrative, health representatives and people will be beneficial in spreading fundamental understanding about the updated Medical Termination of Pregnancy Act, 2021, and safe abortion services to the grass-roots level.

### Strengths and limitations

The current study has several merits: First, this is the first study to contextualize factors that contribute to unsafe abortion in urban and rural settings. Second, this study systematically examines the similarities and differences between pre-defined factors for healthcare utilization by Andersen and the Indian context. These will be useful for researchers and policymakers to formulate more lenient abortion legislation and healthcare coverage with considering the prevalent risk factors for unsafe abortion in rural and urban India.

Despite having certain advantages, the study also had significant drawbacks. First, the extent of unsafe abortion practices may differ because the data was gathered through self-reporting. Second, the study is restricted to capturing causal relationships between outcome and explanatory variables because of the cross-sectional structure of the data. The current study did not consider other individual-level factors such as self-reluctance to use safe abortion treatments due to stigma, in-law’s disapproval, and societal factors. Finally, selection bias in the study sample may impact the outcomes.

## Conclusion

The present study found significant rural-urban divides in unsafe abortion practices based on socio-demographic status. Young women were the most vulnerable regarding unsafe abortion practices in India, particularly in urban settings. The geographical disparity in unsafe abortion practice was found noticeable in India, suggesting that geographical region is an important predisposing factor of reproductive healthcare utilization. Wealth status and unmet need for family planning were found to be enabling and need factors for unsafe abortions in rural India. The present study suggests that there is a need for multi-sectorial programs to reach the target groups with high-unsafe abortion practices. Furthermore, government intervention should also focus on promoting exposure to the local mass media to enhance knowledge and understanding of safety measures for pregnancy termination.

## Data Availability

The dataset analysed during the current study are available in the Demographic and Health Surveys (DHS) repository, https://dhsprogram.com/data/available-datasets.cfm.

## Abbreviations

OR: Odds ratio
IIPS: International Institute for Population Sciences
NFHS: National Family Health Survey
MTP: Medical Termination of Pregnancy
SCs/STs: Scheduled tribes and castes
OBCs: Other Backward Classes
CI: Confidence interval
